# A Developmental Learning Approach of Mobile Manipulator via Playing

**DOI:** 10.3389/fnbot.2017.00053

**Published:** 2017-10-04

**Authors:** Ruiqi Wu, Changle Zhou, Fei Chao, Zuyuan Zhu, Chih-Min Lin, Longzhi Yang

**Affiliations:** ^1^Fujian Provincal Key Lab of Brain-Inspired Computing, Department of Cognitive Science, School of Informatics, Xiamen University, Xiamen, China; ^2^Department of Computer Science, School of Computer Science and Electronic Engineering, University of Essex, Colchester, United Kingdom; ^3^Department of Electrical Engineering, Yuan Ze University, Tao-Yuan, Taiwan; ^4^Department of Computer and Information Sciences, Faculty of Engineering and Environment, Northumbria University, Newcastle upon Tyne, United Kingdom

**Keywords:** developmental robotics, mobile manipulator, robotic hand-eye coordination, neural network control, sensory-motor coordination

## Abstract

Inspired by infant development theories, a robotic developmental model combined with game elements is proposed in this paper. This model does not require the definition of specific developmental goals for the robot, but the developmental goals are implied in the goals of a series of game tasks. The games are characterized into a sequence of game modes based on the complexity of the game tasks from simple to complex, and the task complexity is determined by the applications of developmental constraints. Given a current mode, the robot switches to play in a more complicated game mode when it cannot find any new salient stimuli in the current mode. By doing so, the robot gradually achieves it developmental goals by playing different modes of games. In the experiment, the game was instantiated into a mobile robot with the playing task of picking up toys, and the game is designed with a simple game mode and a complex game mode. A developmental algorithm, “Lift-Constraint, Act and Saturate,” is employed to drive the mobile robot move from the simple mode to the complex one. The experimental results show that the mobile manipulator is able to successfully learn the mobile grasping ability after playing simple and complex games, which is promising in developing robotic abilities to solve complex tasks using games.

## 1. Introduction

Intelligent robots have been widely applied to support or even replace the work of humans in many social activities, such as assembly lines, family services, and social entertainment. These robots are made intelligent by many methods proposed in the literature, with the most common ones being mathematical modeling and dynamics models, such as Yan et al. (2013), Galbraith et al. (2015) and Grinke et al. (2015). These methods utilize predefined cognitive architectures in the intelligent systems, which cannot be used for significant changes during the interaction within the environment. If the intelligent system is applied in a new environment, the intelligent systems must be reconstructed. Also the complexity of the model increases exponentially as the complexity of the task increases. In addition, it is still very challenging in the field of robotics to allow the robot to learn complex skills and incorporate a variety of skills in an intelligent system.

Asada et al. (2001), Lungarella et al. (2003), and Weng (2004) attempt to let the robot learn intricate skills using the so-called developmental robotics approaches. These approaches enable robots to gradually develop multiple basic skills and thus learn to handle complex tasks (Berthouze and Lungarella, 2004; Jiang et al., 2014). In other words, the learning target of a complex set of skills are divided into the learning of a number of stage targets (Wang et al., 2013; Zhu et al., 2015), and the robot achieves the ultimate learning goal by completing a series of sub learning goals. This method reduces the difficulty for the robot to learn new skills (Shaw et al., 2014), and gives the robot the ability to accumulate learning, where the basic skills learned during the development process are reserved so as to arrive at the final skill (Lee et al., 2013). When a robot uses the method of developmental robotics to learn new skills, the target in every developmental phase must be clearly defined (Stoytchev, 2009). However, this is practically very challenge for those phases with a large number of complex tasks, thereby limiting the applicability of developmental robotics.

It has been observed by infant development researchers that infants and young children, when developing skills, do not need to define specific developmental goals (Adolph and Joh, 2007), and mergence is the primary form for infants to acquire skills (Morse and Cangelosi, 2017). In particular, a play phenomenon often accompanies the process of an infant skill development (Cangelosi et al., 2015), which has led to one infant development theory that infants develop relevant skills during play. The play of the early infant is driven primarily by intrinsic motivation (Oudeyer et al., 2007; Baldassarre and Mirolli, 2013; Caligiore et al., 2015), and an infant's development goal is implied in the game that the infant plays. This theory has not been applied and verified in developmental robotics. Therefore, a robotic developmental model that combines the infant developmental theory and developmental robotics is proposed herein. In this model, the learning skills of a robot are artificially viewed as game playing by an infant, and the developmental target is implied in the game goals. Then, a method of developmental robotics is ustilised by the model to accomplish the robot's skill development and learning. The proposed system not only reduces the difficulty of robot learning and allows accumulate learning, but also mitigates the limitation of applicability as discussed above by clearly defining goals in the developmental method.

In contrast to other developmental learning methods (Yang and Asada, 1996; Berthouze and Lungarella, 2004), the proposed approach embeds the role of play in early infant development into the developmental learning approach. Through two game modes, our robot developed mobile reaching and grasping abilities with no external reward existing in the two game modes. The robot merely uses its learning status to switch from one game mode into next one. Such approach also adopts the intrinsic motivation-driven learning method. Therefore, the main contribution of this work is a combined developmental algorithm that allows robot to acquire new abilities by applying the infant developmental theory, in which skills are developed through playing. With the inclusion of game elements, the robot can acquire developed mobile reaching and grasping skills with emergence.

The remainder of this paper is organized as follows: section 2 introduces the background knowledge of developmental robotics and the “Lift-Constraint, Act and Saturate” developmental algorithm. Section 3 outlines our model and designs the developmental strategy of robots. Section 4 describes the experimentation and analyzes the results. Section 5 concludes the paper and points out possible future work.

## 2. Developmental robotics

As a research method with an interdisciplinary background of developmental psychology, neuroscience, computer science, etc. (Earland et al., 2014; Law et al., 2014a; Gogate, 2016), developmental robotics aims to provide solutions in the design of behavior and cognition in the artificial intelligence systems (Marocco et al., 2010; Baillie, 2016; Salgado et al., 2016). Developmental robotics is inspired by the developmental principles and mechanisms observed during the development of infants and children (Chao et al., 2014a), and thus the main idea of developmental robotics is to let a robot imitate a human's development process (Adolph and Joh, 2007; Oudeyer, 2017). The robot achieves sensory-movement and cognitive ability of incremental acquisition according to the inherent development principles and through real-time interaction with the external environment (Cangelosi et al., 2015). Developmental robotics focuses on two primary challenges in the field of robotics: (1) learning new knowledge and skills from a constantly changing environment; and (2) understanding their relationship with their physical environment and other agents.

Guerin et al. (2013) suggested in developmental robotics that most patterns need to be learned from a few patterns and the described knowledge must be developed gradually, by alluding to the general mechanism of sensory-movement development and the knowledge description in action-object relationships. Law et al. (2014a) achieved stage development on an iCub robot. They successfully built a development model for infants from birth to 6 months, which is driven by a new control system. Starting from uncontrolled movements and passing through several obvious stages of behavior, the iCub robot, like an infant, finally reaches out and simply manipulates the object. Cangelosi et al. (2015) used a method of action-centering to perform a large number of synchronous comparisons with similar human development and artificial systems. They discovered that human development and artificial developmental systems share some common practices from which they can learn. These studies inspired the establishment of the proposed robotic systems reported in this paper using the key features and important theories in human infant development.

One of the two most important research focuses in the field of developmental robotic is the development of skills corresponding to a particular stage of an infant's development (Chao et al., 2010; Law et al., 2013), and another is the modeling of the multi-stage development process (Hülse et al., 2010; Law et al., 2014b). However, it is also of significant importance to study the impact of play in early infant development, which may also provide solutions in developmental robotics. Hart and Grupen (2011) proposed a robot which organizes its own sensory-movement space for incremental growth. Their solution uses internal incentive motivations to allow robots to assimilate new skills which are learned from the new learning phase or the new environment and become the basis for the next learning phase. This research has been applied to humanoid robots. Benefiting from important theories on developmental psychology, those humanoid platforms can easily reproduce several behavioral patterns or validate new hypotheses. Savastano and Nolfi (2013) used a neural robot to imitate an infant's learning process, which was demonstrated by a humanoid robot incrementally learning the ability to grasp. In the experiment, the maturity limit was systematically controlled and a variety of developmental strategies were produced. The experiment also shows that human and robots can learn from each other by a comparative study. Different with these studies, the experiment platform presented in this work is a wheeled mobile robot, aiming to study the influence of play in developmental learning methods.

The “Lift-Constraint, Act and Saturate” (LCAS) approach (Lee et al., 2007), as a developmental learning algorithm, has been widely applied in the developmental robot system (Chao et al., 2013; Wang et al., 2014). The LCAS approach contains a loop with three segments: (1) Lift-Constraint, (2) Act, and (3) Saturate. First, all possible (or available) restrictions are stated clearly and their release times are formulated. Then, the robot learns that all existing constraints are substantiated. When the saturation rate of a robot's learning system is stable, a new constraint is released. From this, the robot learns either new knowledge or skills leading to the removal of a new environmental constraint. When the robot has lifted all constraints through learning, and all saturation rates of the robot learning system are stable, the robot has successfully learned a series of skills.

## 3. The proposed method

### 3.1. Model overview

A developmental algorithm is proposed in this work by designing a game for robots that allows robots to develop skills by playing. Infants' stable grasping abilities are developed through grasping objects around them and infants are not happy until they can stably do so. Due to the constraints of body, infants pay most of their attentions on the range of physical activities while learning skills, which leads to more efficient skill learning. In the process of learning, infants do not have a clear learning goal, and all the activities are simply driven by intrinsic motivation and interest. Inspired by this, in our model, we design a game of pick-up-toys for a mobile manipulator, in which victory is the mobile manipulator successfully picking up surrounding toys. By playing this game, a robot with a mobile manipulator gradually develops mobile grasping ability. The game has two modes based on task complexity:

- **Simple game mode:** All toys are distributed within the working range of the manipulator, and the robot can perform toy grasping without moving.
- **Complex game mode:** Some toys are distributed beyond the working range of the manipulator, and the robot must move in order to grasp these toy.

**Figure 1:**
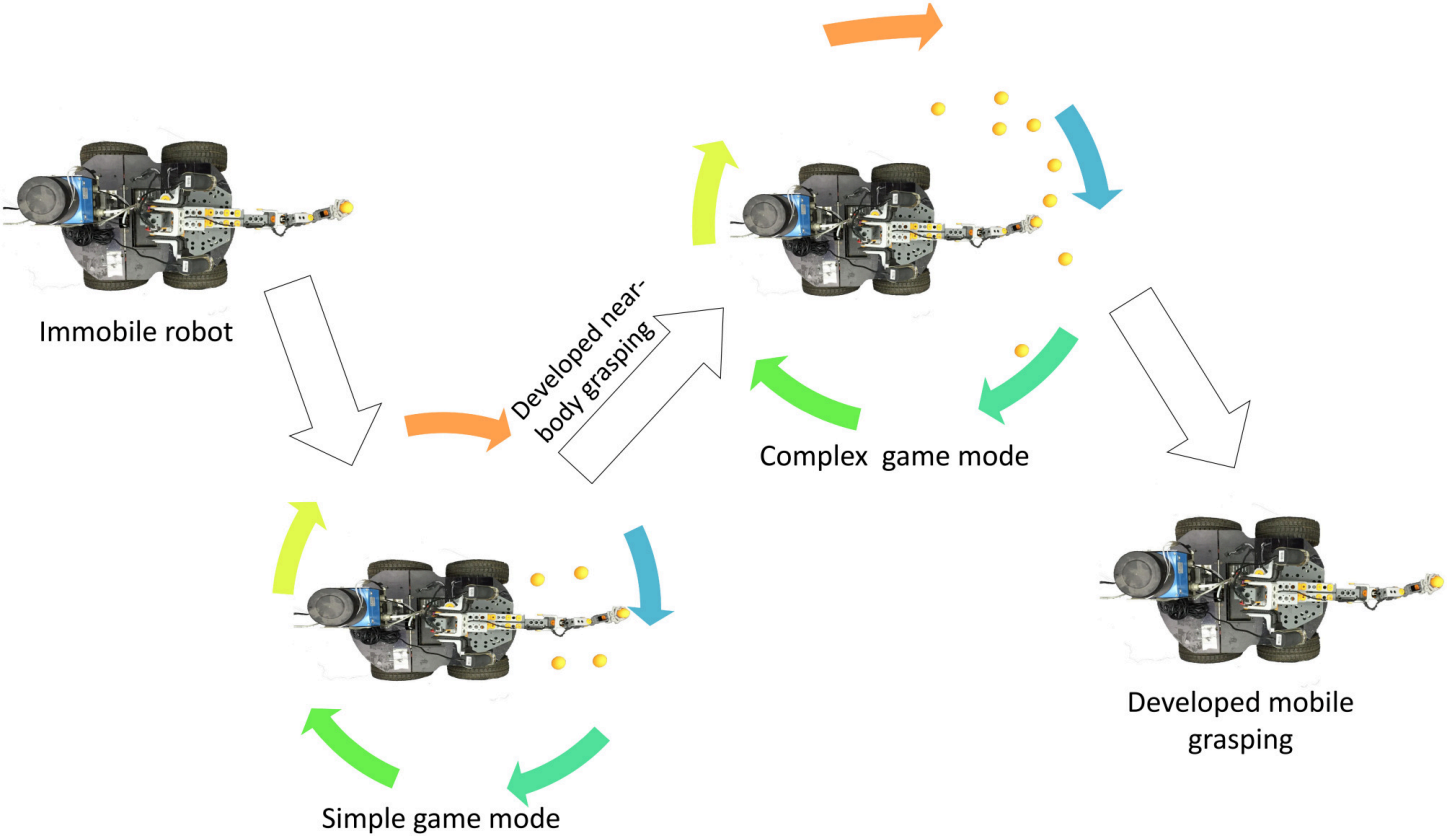
The whole procedure of the game.

The robot's skill development process is illustrated in Figure 1. The robot with the mobile manipulator is initialised to play in the simple game mode until it successfully completes the game. After the robot acquires near-body grasping ability, the game switches to the complex mode. The game is over when the robot acquires mobile grasping ability in this game mode.

The key in implementing a mobile robot with grasping ability is the coordination of the visual system, the manipulator, and the mobile system. This is mainly implemented by two basic non-linear mappings: (1) from the robot's visual space to the manipulator's movement space, and (2) from the visual space to the robot's mobile space. The two basic mappings are simulated in this work by two Radial Basis Function networks (RBF), and the training of these neural networks is accomplished by the robot playing the game.

### 3.2. Developmental strategy

Before acquiring mobile grasping ability, infants must attain a number of developmental stages as listed in Table 1 (Law et al., 2010, 2011), in which they develop a variety of basic abilities. A developmental strategy is designed to support the development of a robot's mobile grasping ability, which is implemented by the LCAS algorithm (i.e., Algorithm 1 shown below) (Chao et al., 2014b). In this pseudo code, *i* is the number of learning epochs under current constraints, and *Sat*(*i*) is the saturation rate in the *i*^*th*^ learning epoch. If the *Sat*(*i*) is true, the algorithm ends the training under the current constraint, and releases a new constraint. The value of *Sat*(*i*) is determined by Equation 1, where *i* is the number of training epochs; *G*(*i*) is the model's global excitation value at epoch *i*; ϵ controls the sampling rate; and the ϕ is a fixed value used to control the global excitation's amplitude of variation. If the value of *G*(*i*) is < ϕ and the variation of the global excitation is < ϵ, a new constraint is lifted. In this work, the values of parameters ψ, ϵ and ϕ are empirically set to 10, 0.5, and 0.02, respectively, and theoretical study on these parameters remain as future work.

(1) $$Sat(i)=\{\begin{array}{ccc} true; & if & |G(i)-G(i-\psi )|<\epsilon \\ & and & G(i)<\phi ;i=\psi \cdots n\\ false; & else & \end{array}$$

**Table 1:** Infant's developmental stages and the corresponding development abilities.

**The developmental stage**	**Developed ability**
Visual fixation period	Fixation, saccade
Hand-eye coordination period	Hand-eye coordination, near-body grasping
Mobile-direction coordination period	Mobile grasping

**Algorithm 1:** Combined Learning Algorithm

1: **while** not all constraints are released **do**
2: **for** *i* = 0 to *n* **do**
3: **if** *Sat*(*i*) is TRUE **then**
4: quit this for-loop and release a new constraint;
5: **else**
6: repeat doing the leaning process within this for-loop (Lines 2-8);
7: **end if**
8: **end for**
9: **end while**

In the LCAS algorithm, a robot's constraints are first substantiated, as shown in Table 2. Once the constraints are substantiated, the development of the robot proceeds according to the lift-constraint strategy as listed in Table 3.

**Table 2:** The constraint instantiation for mobile robot.

**Constraint type**	**Substantiation**
Hardware	Eye joint, arm joint, wheel motor
Sensory-motor	Tactile sensor, arm movement range
Cognitive	Neural network
Maturational	Network convergence threshold
External/environmental	Location of the target object

**Table 3:** The robot's lift-constraint strategy.

**Lift sequence**	**Developed ability**
1. Visual resolution	Fixation ability
2. Eye joint	Saccade ability
3. Shoulder joint, Elbow joint	Hand-eye coordination
4. Wrist joint, gripper joint	Near-body grasping
5. Wheel joint	Mobile grasping

The LCAS algorithm in this work is executed in the following five steps: (1) The target object is placed in the robot's external environment. The robot acquires image information about the environment by removing the “visual resolution” constraint of the robot's eyes. (2) After the robot attains watch ability, the eye joint constraint is lifted. The robot learns saccade ability by eye joint movement. (3) Then, the arm joint constraint is lifted to allow for the movement and sensory abilities of the robot's arm. After the motor babbling stage, the robot builds hand-eye coordination. Accordingly, the robot executes the reaching action. (4) From this, the tactile sensor constraint in the arm is removed after the robot builds hand-eye coordination. Based on hand-eye coordination, the robot detects whether the object in the gripper uses the tactile sensor. At this stage, the robot learns near-body grasping. (5) Finally, the wheel joint constraint is removed, and the robot has mobile ability. Then, the robot learns mobile grasping by building the mapping between visual and mobile space.

**Figure 2:**
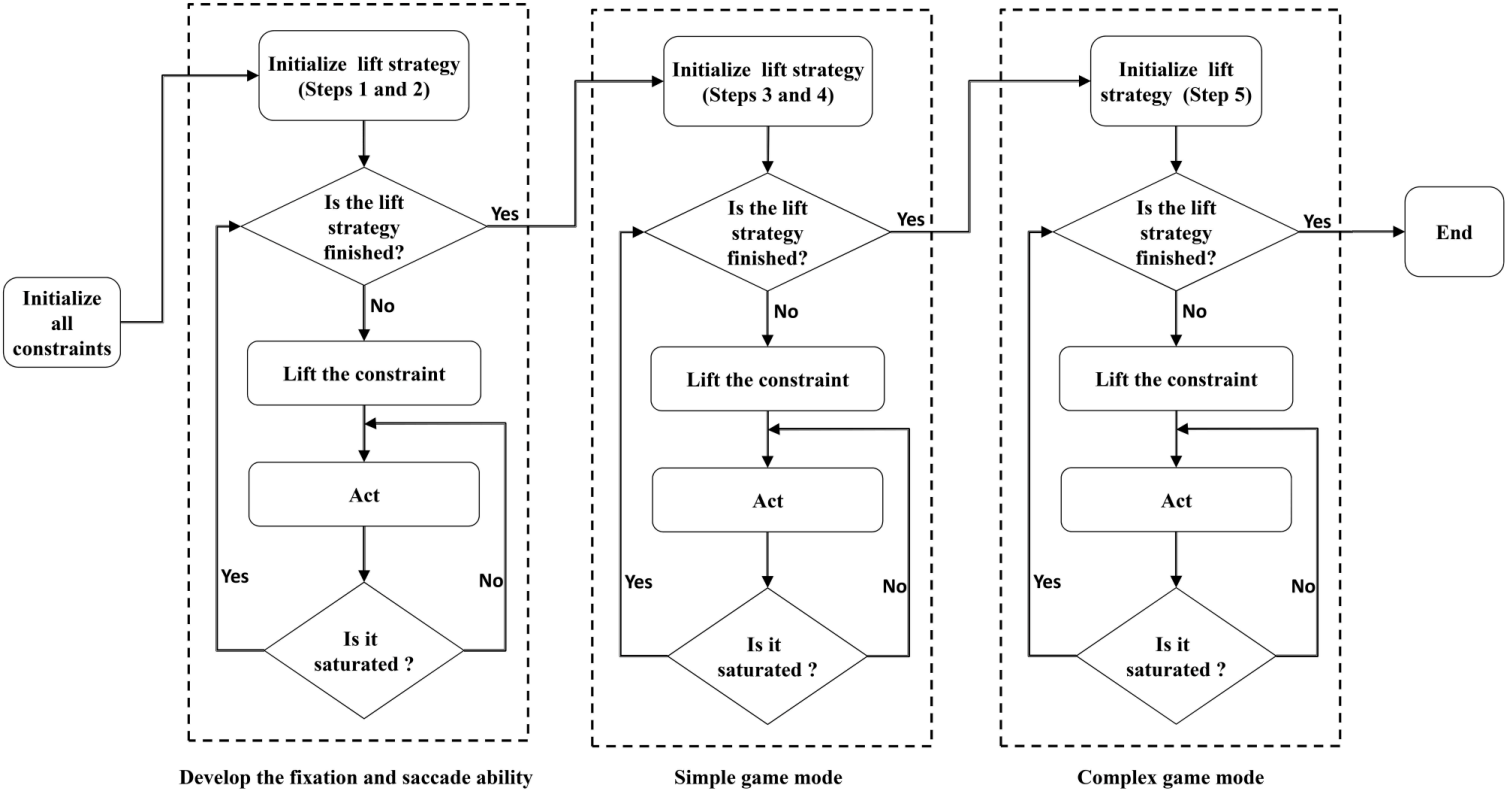
The LCAS algorithm implementation and the overall training processes.

After the first two steps of training, the robot developed fixation and saccade abilities, and thus the robot can play the simple game (Chao et al., 2016). In the simple game mode, the third and fourth steps of the lift-constraint strategy are executed, and the robot develops hand-eye coordination and near-body grasping. The fifth step of the strategy is executed in the complex game mode, where the robot develops mobile grasping ability. The entire procedure for model training and execution is illustrated in Figure 2.

### 3.3. Mobile robot hardware system

**Figure 3:**
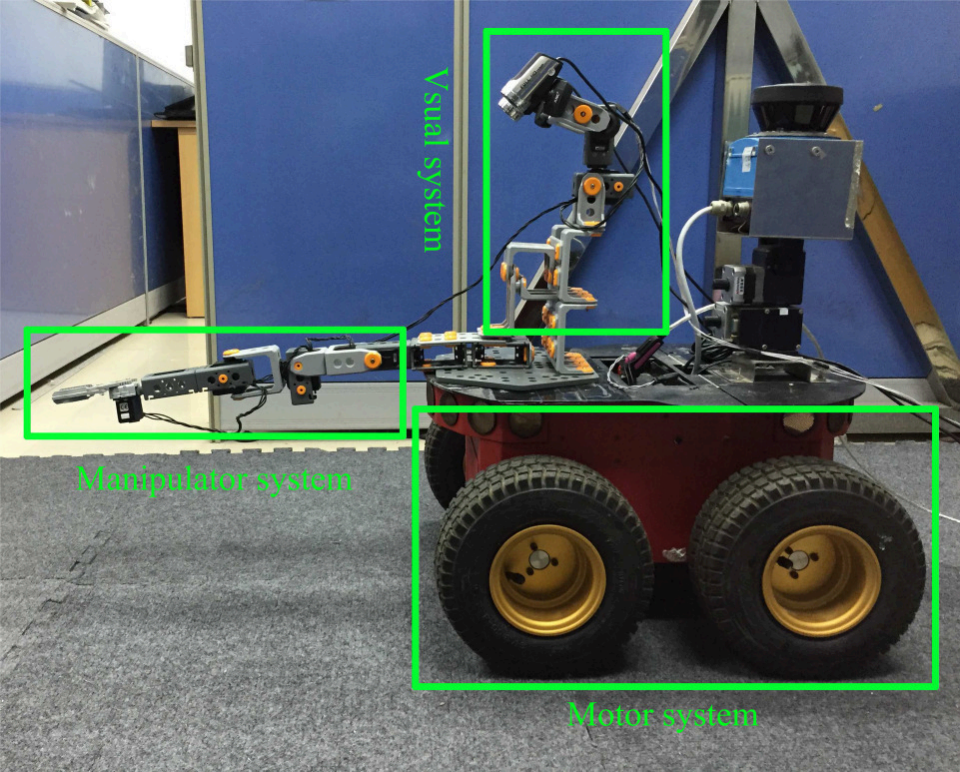
The robotic hardware.

The mobile robot's intelligent system is mainly composed of three subsystems: the visual subsystem, the manipulator, and the motor, as demonstrated in Figure 3. The detailed functions of the three systems discussed below.

1. **The visual subsystem**. The robot's visual system performs two tasks including finding the object and locating the object. Firstly, the visual system analyzes the image color information captured from the robot's two eyes, and detects whether the target is in the field of view. Secondly, if the target is in the field of view, the visual system, through fixation ability, acquires the retinal position of the target, *S*(*x*_1_, *y*_1_, *x*_2_, *y*_2_), wherein *s*_*l*_(*x*_1_, *y*_1_), and *s*_*r*_(*x*_2_, *y*_2_) express the coordinates of the target in the left and right eyes, respectively. The combination of the left and right eyes represents the target retinal position. Finally, the visual system, using the saccade ability, combines the retinal coordination, *S*(*x*_1_, *y*_1_, *x*_2_, *y*_2_), and the eye joint, *S*_*h*_(*j*_5_, *j*_6_), to generate the visual space coordination, *P*(γ, θ).
2. **The manipulator subsystem**. The physical structure of the manipulator system simulates the human arm structure. It contains four joints, denoted as (*j*_1_, *j*_2_, *j*_3_, *j*_4_). Its first three joints (*j*_1_, *j*_2_, *j*_3_) correspond to the human arm joints (shoulder, elbow and wrist), respectively. These three joints construct the movement space of the robot arm. The *j*_4_ joint represents the gripper of the manipulator and is used to simulate the grasp ability of the human palm.
3. **The motor subsystem**. The motor system consists of four wheels, which enable the robot to execute mobile actions, such as forward motion, backward motion, and turns. Because only the front two wheels of the mobile robot have a motor, the robot's moving motor is denoted as *M*(*m*_1_, *m*_2_). The robot controls its movements by changing the value of *M*(*m*_1_, *m*_2_).

### 3.4. Game processes

#### 3.4.1. Simple game process

The simple game mode requires the robot to pick up balls that are scattered within the work range of the manipulator. In this game mode, the robot's eyes only focus on the work range of the manipulator. After it implements the first two steps of the lift-constraint strategy and develops fixation and saccade abilities, the robot can participate in the simple game mode, as shown in the center frame of Figure 2. When the robot plays the simple game, it must build a mapping from the visual space to the manipulator's movement space. This mapping is simulated by one of the two RBF networks, *R*_1_. Different from the experiment hardware used by Caligiore et al. (2014), the robot is not a humanoid robot in this work. Therefore, it is impossible to use real data collected from infants, and instead the training samples are re-collected based on the wheeled robot. The training sample of this network consists of the visual space coordination, *S*_*h*_(*p*, *t*), and the manipulator's joint value, (*j*_1_, *j*_2_, *j*_3_, *j*_4_). In the RBF network, the center positions of the RBF neurons are determined by a K-means algorithm, and the number of RBF neurons is set to twice the number of input dimensions. In this work, the Gaussian kernel is applied in the radial basis function. The calculation of the RBF network is shown in Equations (5) and (6), where *y*(*x*) denotes the network's output joint value, and *w_i_* denotes the weights of the hidden layer, ϕ_*i*_ denotes a radial basis function, δ denotes the width of the Gaussian kernel, and it is empirically set at $\sqrt{5}$.

(2) $$y(x)=\sum_{i=1}^{i}w_{i}\phi_{i}$$

(3) $$\phi_{i}(x)=exp(-\frac{\|x-x_{i}{\|}^{2}}{2\delta^{2}}),\delta >0$$

In the process of training the robot's hand-eye coordination, a yellow ball is placed as a target object in the gripper of the manipulator. The ball moving randomly with the manipulator, and the sample *e*(*P, M*_*a*_) are collected in each movement. The sample obtained is placed in the sample pool, *E*(*e*_1_, *e*_2_, *e*_3_, …, *e*_*n*_). When the number of samples in the sample pool reaches a fixed number, some samples are randomly selected as the training samples for the *R*_1_ network. After that, the *R*_1_ network is trained using the Backpropagation algorithm. During the collection of the sample, the values of the wrist, *j*_3_, and the gripper, *j*_4_, are fixed, because the shoulder *j*_1_ and the elbow *j*_2_ already represent most of the movement space of the robot, while the wrist, *j*_3_, is more involved in the grasping action (Marini et al., 2016). When the *R*_1_ network training is saturated, the robot develops a basic hand-eye coordination capability, and the wrist, *j*_3_, and the gripper, *j*_4_, can be released. After this constraint is removed, the samples in the sample pool must be re-collected. The *R*_1_ network uses these newly collected samples for further training, and, finally, the robot develops near-body grasping ability.

#### 3.4.2. Complex game process

When the learning status is stable in the simple mode, the robot switches to the complex game mode using whole field view. The procedure for the complex mode is shown in the right-hand box of Figure 2. In the complex mode, the balls are scattered within the visual range of the robot, but not in the work range of the manipulator. To pick up these balls, the robot must relocate itself. The mapping relationship between the visual space and the robot's mobile space is built in the complex mode. This mapping is simulated by another RBF neural network, *R*_2_. The training method and the set of parameters are the same as those used in the *R*_1_ network.

The training samples of the *R*_2_ network are based on two sequences: (1) the movement trajectory of the target in the robot's visual space, *PS*(*p*_*s*1_, *p*_*s*2_, …, *p*_*st*_), and (2) the variation sequence of the robot's moving motor value *MS*(*M*_1_, *M*_2_, …, *M*_*t*_). In *PS*, *p*_*st*_ denotes the coordinate distance of the ball in the robot's visual spaces when the robot moves from step *t* to step *t* + 1. The values of *p*_*st*_ is determined by Equation (4), where *p*_*t*_(γ, θ) denotes the position of the target in the visual space at step *t*, and *p*_*t*+1_(γ, θ) denotes the position at step *t* + 1. Likewise, the change of the motor value from step *t* to step *t* + 1 is represented as *M*_*t*_. So, the former n-step movement trajectory and the accumulated change of the motor are expressed by Equations (5) and (6) respectively, where *PA*_*n*_ denotes the accumulated distance from the target to the robot when the robot moves *n* steps, and *MA*_*n*_ denotes the accumulated change of the motor.

(4) $$p_{st}=p_{t+1}(\gamma ,\theta )-p_{t}(\gamma ,\theta )$$

(5) $$PA_{n}=P_{n+1}\;-\;P_{1}$$

(6) $$MA_{n}=\sum_{n=1}^{n}M_{n},\;n\leq D$$

In the complex game, a target is placed within the field of the robot's vision, rather than within the manipulator's work field. Then, the robot is set to randomly move *n* steps. If the ball enters the manipulator work field occasionally during the *n* steps, the entire movement trajectory is chosen as a sample. However, if the ball is out of the field of robot's vision during the *n* steps, this trajectory is abandoned, and the target is randomly placed in a new position to start the iteration again.

This stage also requires the following two additional restrictions on the mobility of the robot. (1) Because the accuracy of the robot hardware is limited, the number of mobile steps *n* in a task must be less than the threshold, denoted as *D*. If the number of steps is too big, the accumulated error of the motor will be very large. (2) If the target disappears from the visual field during the robot moving, the task is considered to be a failure, and a new task is started by resetting the game. When the robot reaches the target position within the number of steps, *t*, less than the threshold, the *PA*_*n*_ and the *MA*_*n*_ are combined into a sample *e*(*PA*_*n*_, *MA*_*n*_). The training *R*_2_ network begins after enough samples have been collected in the sample pool *E*(*e*_1_, *e*_2_, *e*_3_, …, *e*_*n*_). After the mapping relationship is properly established by training the *R*_2_ network, the robot develops mobile grasping ability. When performing a mobile grasping task, the robot completes the task in two steps. Firstly, the robot moves toward the target within the work range of the manipulator using the trained *R*_2_ network. Then, the robot uses *R*_1_ to complete near-body grasping.

## 4. Experiments and analysis

### 4.1. The simple game

**Figure 4:**
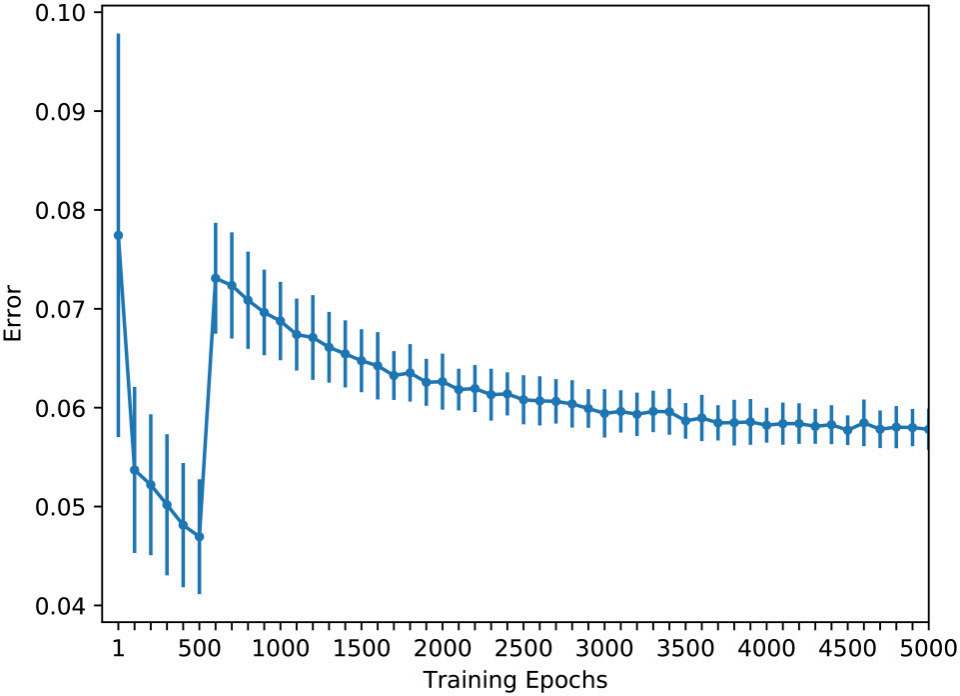
The training of the hand-eye coordination network.

**Figure 5:**
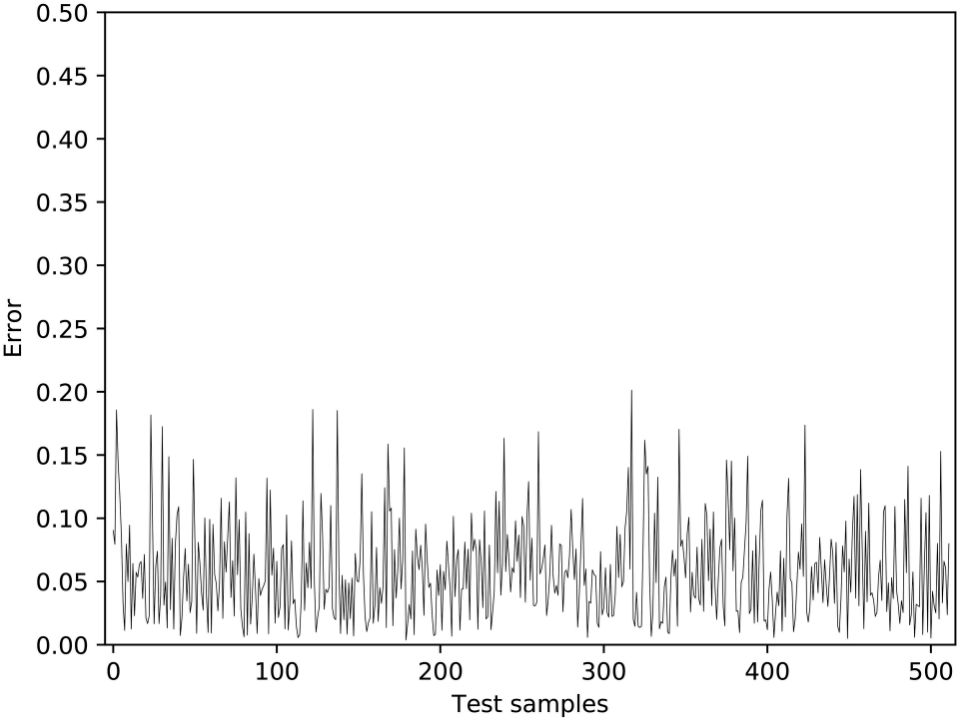
The test results of near-body grasping.

In order to train the hand-eye coordination network, 2,200 training samples and 512 test samples were collected. The error change during the training process is shown in Figure 4. In this figure, each circle denotes the average error after the experiment has been ran for 50 times; and each vertical bar denotes its standard deviation. The training err quickly reduced before the wrist and gripper joints were released. The average error was reduced to <0.05 after just 500 training epochs, which is the point that the robot has successfully learned the hand-eye coordination. Then, the constraints of the wrist and gripper joints were released and the training of the robot's near-body grasping capability was initiated. Figure 4 shows that, in the near-body grasping training stage, the training error begins to decline slowly after a rapid increase, eventually converging to about 0.06. After the network finished training, we tested it with 512 test samples, with the results shown in Figure 5. The overall average error for the 512 tests is about 0.07. Given that it is generally a success if the average error is <0.1 in robot hardware systems, it is clear that the proposed robot has successfully learned the near-body grasping skill.

**Figure 6:**
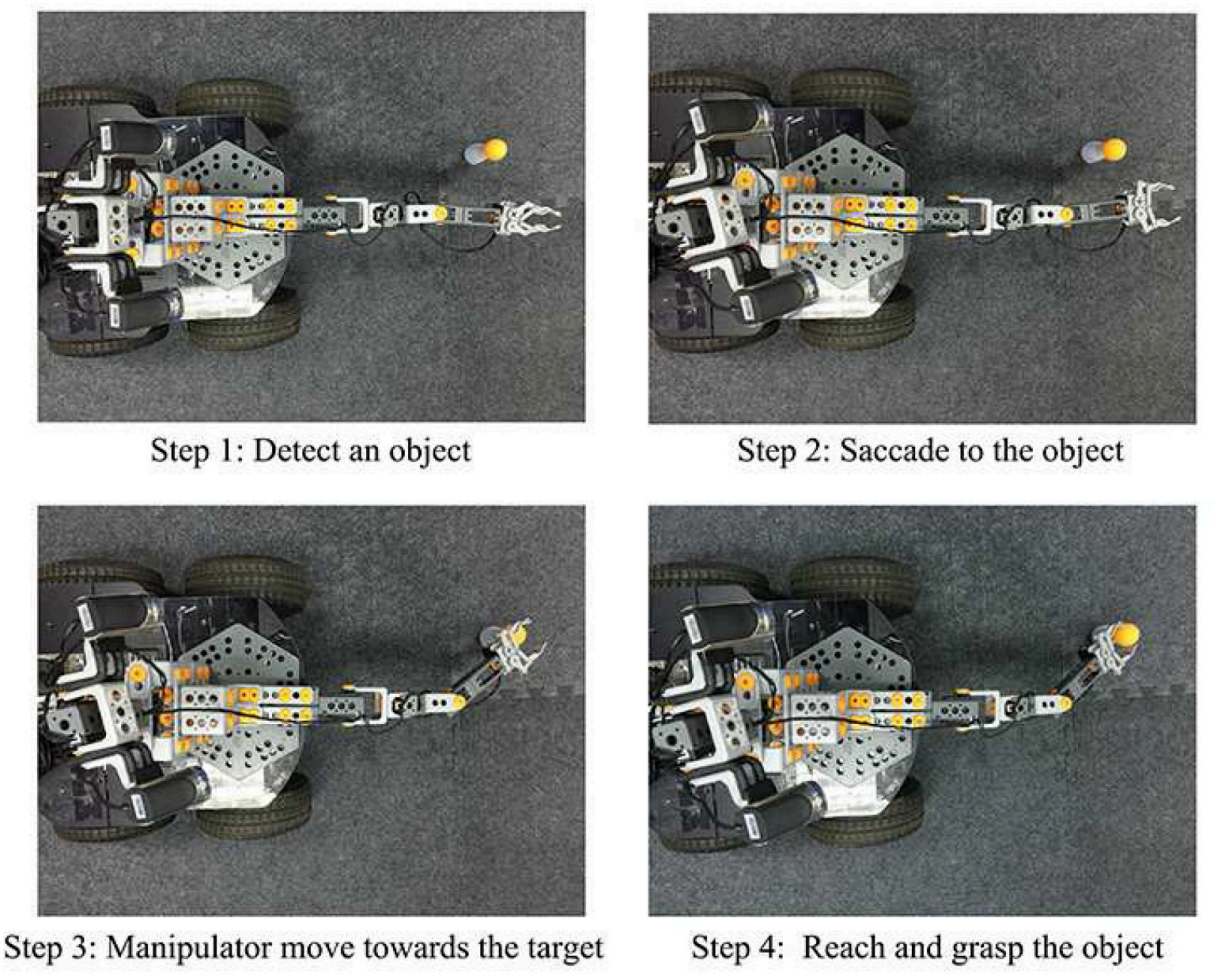
The procedure in simple game mode.

Figure 6 shows the robot's performance during the game after it has learned the near-body grasping skill. In the first step, the robot detects whether the target is within the working range of the manipulator. If the target is within that range, the robot proceeds to the second step, where it views the target, by a saccade and obtains the exact position of the target within its field of vision. After that, the robot maps the visual position of the target into the movement space of the manipulator, and drives the manipulator toward the target position. Finally, the manipulator reaches and grasps the target.

### 4.2. The complex game

**Figure 7:**
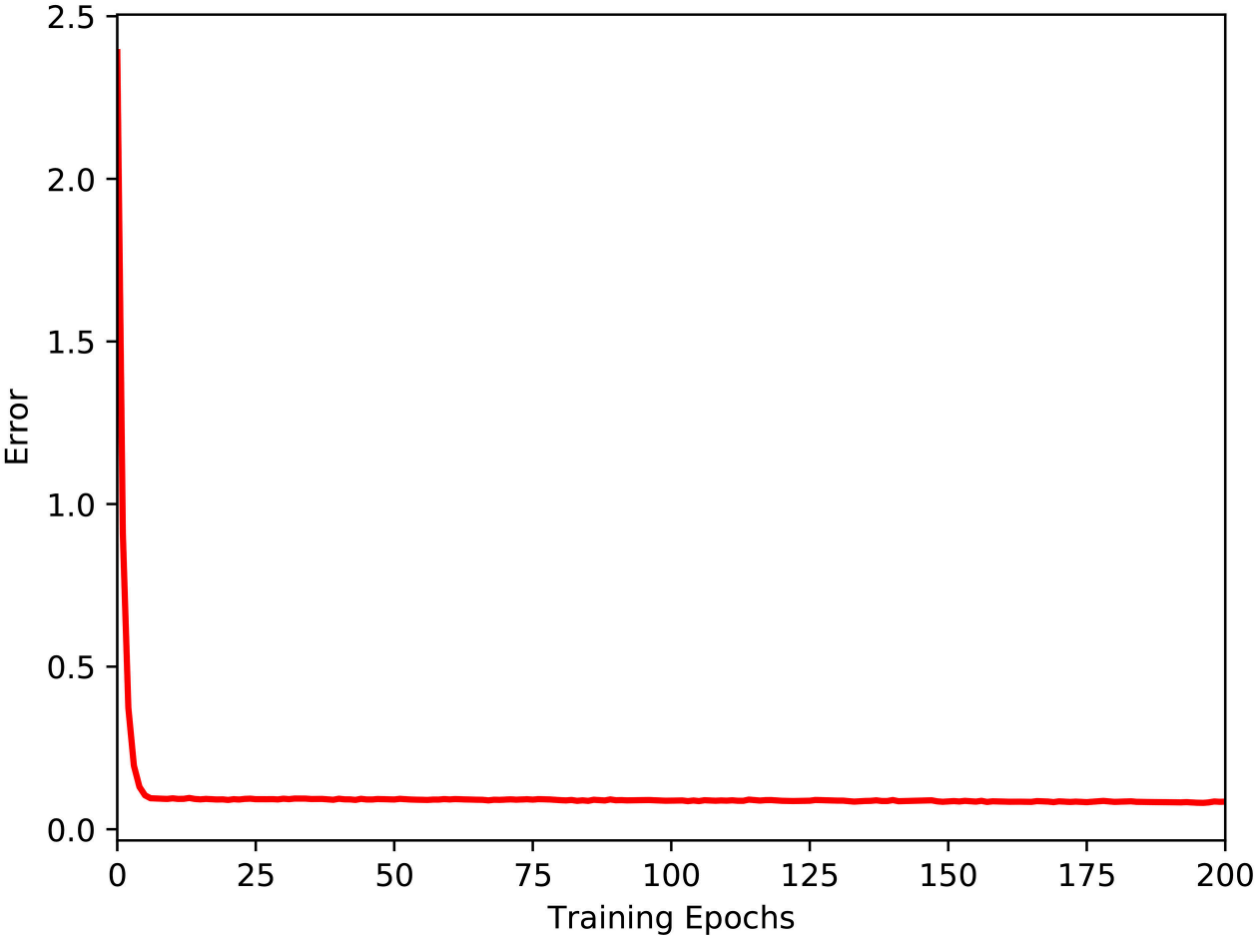
The training of the eye-mobile coordination network.

The eye-mobile network was trained after succesfully trained the hand-eye network. In this stage, 600 samples were collected, of which 500 samples were used for training and the remaining 100 samples for testing. The threshold, *D*, is set as 7. This experiment has also been run for 50 times. Changes of the average error in the training process are shown in Figure 7. As seen in Figure 7, the training error immediately declines rapidly and reaches a stable minimum, indicating that the mapping between the robot's visual and mobile spaces is not very complicated.

**Figure 8:**
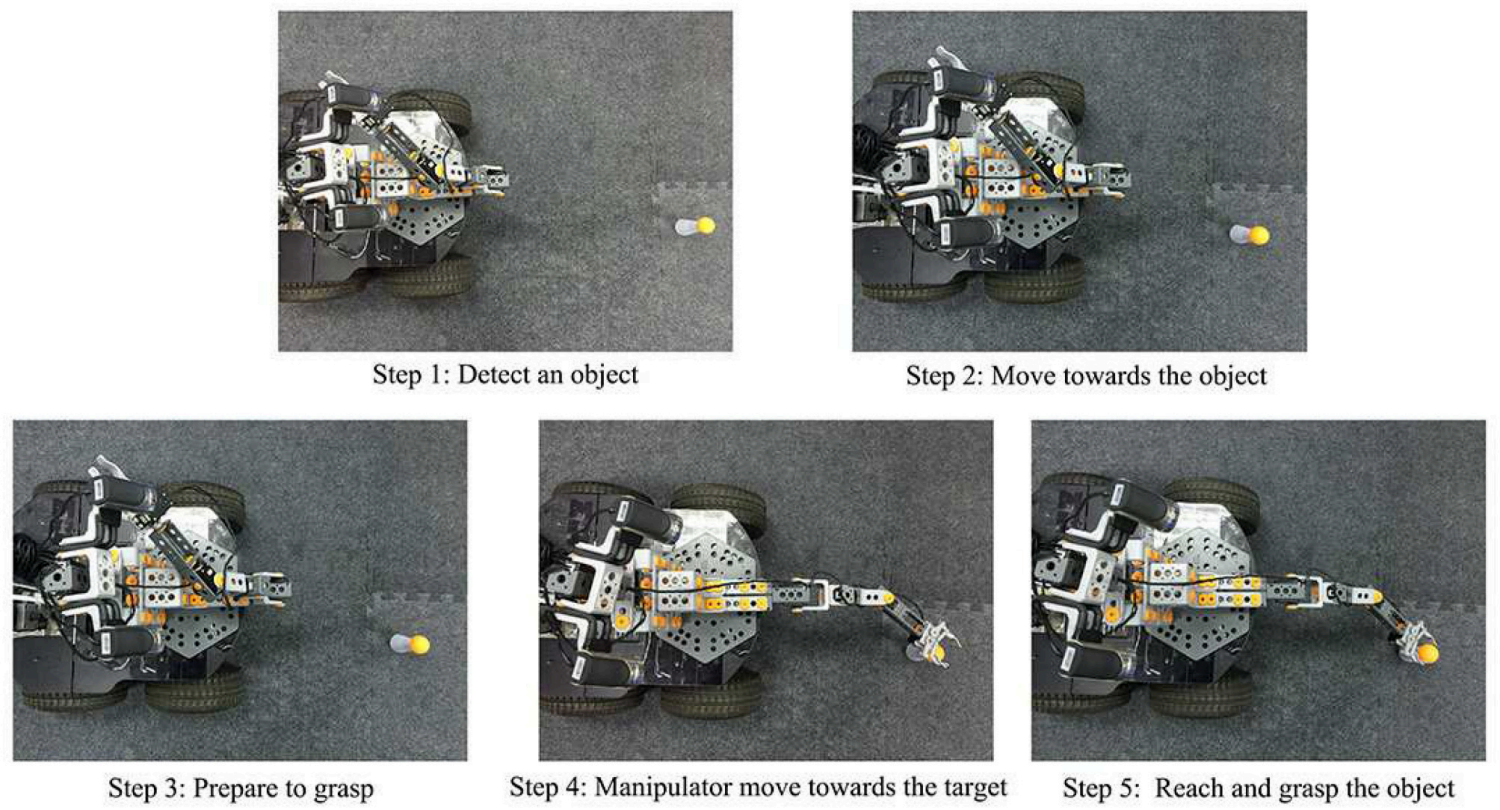
The procedure in complex game mode.

Figure 8 illustrates the robot's performance during the complex game mode after the eye-mobile network has finished training. In Step 1, the robot uses the visual system to obtain the position of target and detect whether the target lies within the working range of the manipulator. In Step 2, if the target is not in the working range of the manipulator, the robot drives the mobile system toward the target position until the target appears in the working range of the manipulator. In Step 3, after the target appears in the working range of the manipulator, the robot stops moving and reacquires the visual position of the target. In Step 4, the robot drives the manipulator toward the target, and in Step 5, the robot reaches and grasps the target.

### 4.3. Performance analysis and comparative study

**Figure 9:**
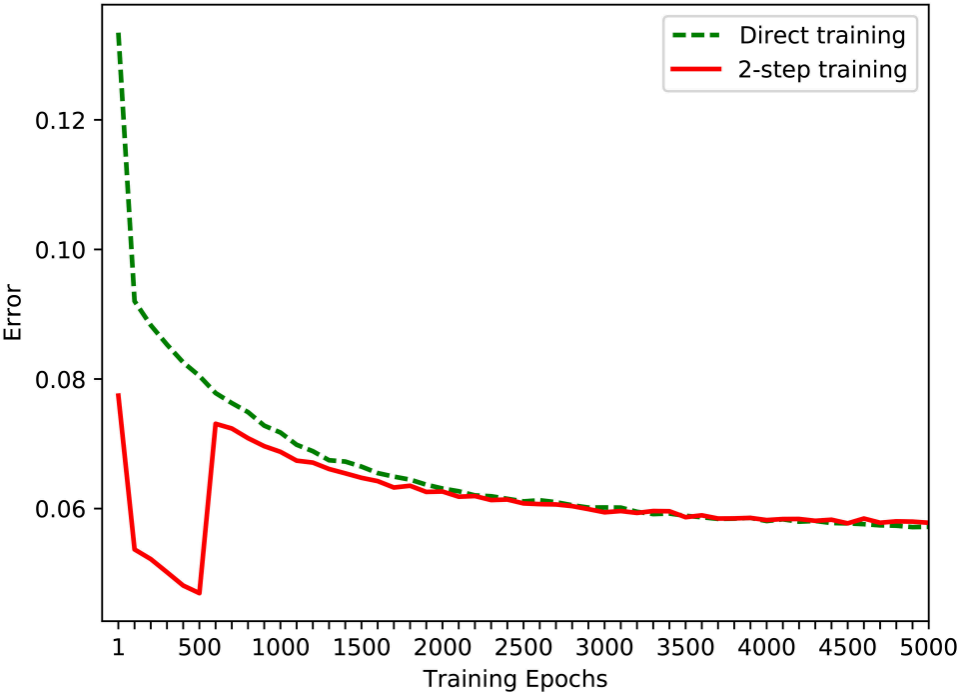
The comparison of two methods in simple game mode.

The experiment discussed above allows the robot, step by step, to learn the near-body grasping skill using a developmental approach. In order to facilitate comparative study, another experiment with the same target of near-body grasping skill was designed using conventional direct training in the simple game mode. The performance of these two methods is compared in Figure 9, where the dotted line is the change of average training error that is learned directly in the simple game mode, and the solid line is the change of average error using the developmental method. As the results shown in Figure 9, in the first 2,000 epochs, the training efficiency of development method is higher than that of the direct training method. After 2,000 training epochs, the training efficiencies of these two methods achieve at a close error value. This phenomena proves that our approach is superior to the conventional method in learning efficiency. In the experiment using the developmental method, in the first 500 epochs, the network was trained using training samples for a robot whose wrist and gripper joints were constrained. After the error is less 0.05, the constraint was removed and the entire range of samples was used to retrain the network. As shown in Figure 9, as the number of epochs increases, the error rate for both the 2-step developmental and direct training approaches decreases. However, over the entire range of epochs, the error decreasing rate of the developmental approach is faster than that of the direct approach, indicating that using the developmental method in a game improves the learning efficiency of a robot.

To summarize, with the experiment of playing game, the robot successfully learned the mobile grasping ability by playing simple and complex games. Through the above experiments, we can conclude the following two results: (1) the proposed approach enables robots to learn skills by modeling the play activities during human infant development. (2) The developmental method with the game elements improves the robot's learning efficiency.

### 4.4. Comparison and discussion

A comparison of Figure 4 with Figure 7 shows that the error rate for the eye-mobile network decreases more rapidly than that for the hand-eye network. However, in the early training epochs, the error rate is higher for the eye-mobile network than for the hand-eye network, because the mobile system has only two joints, but the manipulator has four. Therefore, mapping from visual space to mobile space is simpler than mapping to the manipulator movement space. On the other hand, fewer dimensions also make the output more sensitive to the input values. In Figure 7, the reason of the rapid error decreasing in *R*_2_ is that a simplified motor mode, mapping the visual space to robot's motor position space, is used in this work. The target's visual coordination and the robot's wheel movement trajectory are collected as training samples, in which, the motor mobile value has only two dimensions. The mapping between the robot's visual and mobile spaces is not complicated. However, if the work uses the mobile platform's dynamic control model, which can support the acceleration control for our robot, the network will require more learning time for the more complicated control. In Figure 4 the error rate rises rapidly after the wrist and gripper joints are released at the five hundredth epoch, because the mapping becomes more complex. However, the error rate after the rapid increase is still lower than that for the directly learning approach, proving that the learning in the previous stage is helpful for the next learning stage.

**Figure 10:**
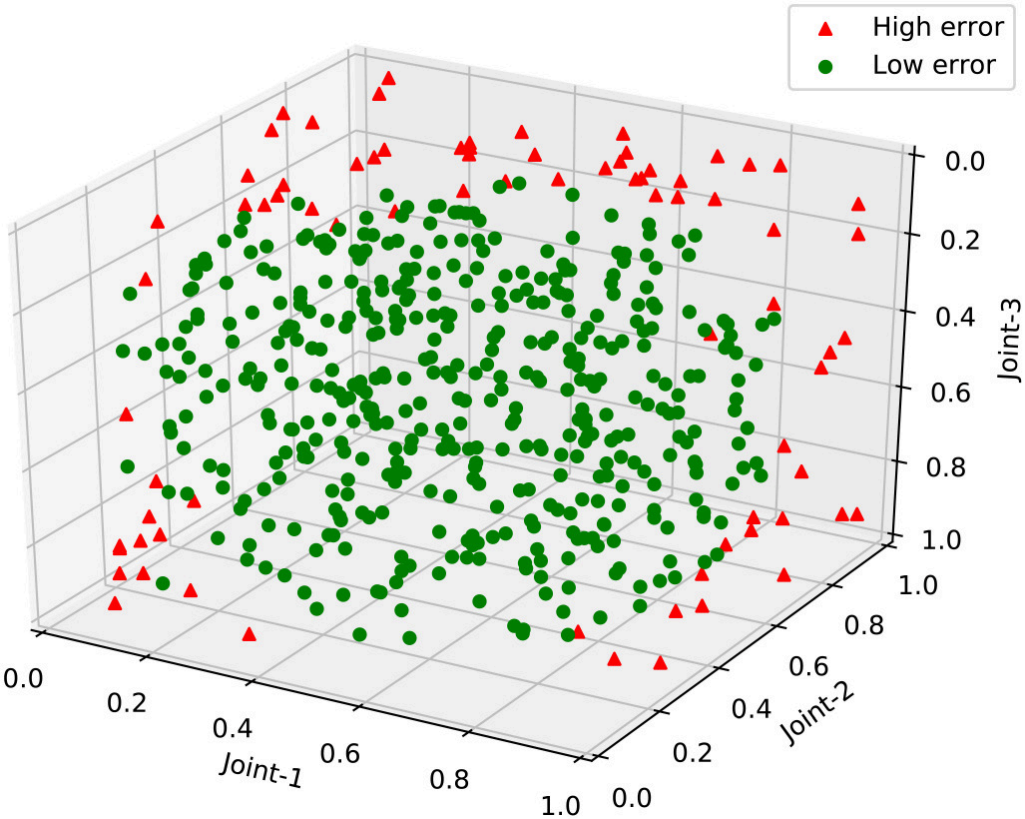
The results of the near-body grasping test.

After testing the robot's near-body grasping ability, we further analyze the test results from the perspective of the manipulator's movement space. Because the gripper joint has only two ways to open and close, it has little effect on the variation of the manipulator in the movement space. Therefore, we analyze just the first three joints of the manipulator. The analysis results are shown in Figure 10. Triangles represent the test sample for which the error rate is >0.1; circles represent the others. Figure 10 shows that most of the high error actions have at least two joints and an angle value near the extremum. In other words, these actions, distributed around the edge of the movement space, may be due to the instability of control when the manipulator's servos are near the maximum and minimum angles. Instead, the robot's grasping errors are generally below 0.1 at all other places. By removing the hardware factor, we assume that the robot has built the mapping from the visual space to the manipulator movement space.

For comparison, many developmental models used humanoid robots as experimental platforms (Marocco et al., 2010; Shaw et al., 2014; Morse and Cangelosi, 2017) in particular, several of them are infant-like robots. Those humanoid platforms directly benefit from important theories on developmental psychology, so that these platforms can easily reproduce several behavioral patterns or validate new hypotheses. In contrast, the shape and configuration of a mobile manipulator are very different from those of a human; therefore, the developmental theories need to be adjusted to fit the robot system. However, currently, the mobile manipulators are competent to practical applications, which also requires the robots to have cognitive abilities. Thus, new developmental theories validated by mobile manipulators can be rapidly applied in real-life applications. Moreover, our work focus on using the “Play” strategy to create the mobile reaching ability for our robot, with the two game modes created. Our system not only involves spontaneous and intrinsically motivated exploration of actions and objects in varying contexts (Lee, 2011) but also, contains developmental characteristic by applying the “LCAS” developmental learning algorithm. Without setting specific goals, the robot uses its learning status to develop from the simple game mode to the complex one. The combination of developmental robotics and play modeling leads our robot to have faster learning rate.

## 5. Conclusion

Scientists have found that infants develop a number of skills when playing games in infant developmental research. In this paper, we combined these infant development theories with the LCAS algorithm to generate a developmental algorithm, which does not require specifically defined developmental goals for a robot. We designed two game modes and two RBF neural networks to simulate the procedures necessary for a robot to play in these game modes, with the support of a developmental strategy. The experiments demonstrated that a robot successfully learned moving and grasping skills. From results analysis and comparison, it can be concluded that: (1) a robot can successfully learn near-body grasping and moving grasping skills through play, and (2) in regard to a robot learning these skills, the developmental approach reduces the complexity and accelerates the learning speed.

Our model also has some limitations which can be mitigated in the future. For instance, in order to implement the hand-eye coordination system rapidly, our model uses an open-loop method, which may lead to several failed grasping. In addition, our model does not use the data obtained from real infants. In order to address these, a close-loop method may be used to improve the success rate of grasping. In addition, as an infant develops skills through play, the infant's intrinsic motivation and ability to imitate are closely related to that play (Santucci et al., 2013; Oudeyer et al., 2016). In other words, infants achieve unsupervised learning in their environment through intrinsic motivation, which plays an important role in the control of the infancy learning stage transformation. However, infants learn new skills faster than other babies if they have a strong ability to imitate during the learning process. Therefore, the applicability of the proposed system can be extended in the future by incorporating intrinsic motivation and ability.

## Author contributions

RW performed the experiments and wrote the manuscript; ZZ and FC designed the robotic learning approach; CZ provided psychological analysis on the experimental data; CL designed the robotic control system; and LY analyzed the experimental results and edited the manuscript.

### Conflict of interest statement

The authors declare that the research was conducted in the absence of any commercial or financial relationships that could be construed as a potential conflict of interest.
